# Virtual reality in the treatment of persecutory delusions: randomised controlled experimental study testing how to reduce delusional conviction

**DOI:** 10.1192/bjp.bp.115.176438

**Published:** 2016-07

**Authors:** Daniel Freeman, Jonathan Bradley, Angus Antley, Emilie Bourke, Natalie DeWeever, Nicole Evans, Emma Černis, Bryony Sheaves, Felicity Waite, Graham Dunn, Mel Slater, David M. Clark

**Affiliations:** **Daniel Freeman**, PhD, DClinPsy, **Jonathan Bradley**, DClinPsy, Department of Psychiatry, University of Oxford, Oxford, UK; **Angus Antley**, PhD, Department of Psychiatry, University of Oxford and Oxford Health NHS Foundation Trust, Oxford, UK; **Emilie Bourke**, ScM, **Natalie DeWeever**, BSc, **Nicole Evans**, BSc, **Emma Černis**, MSc, **Bryony Sheaves**, DClinPsy, **Felicity Waite**, DClinPsy, Department of Psychiatry, University of Oxford, Oxford, UK; **Graham Dunn**, PhD, Centre for Biostatistics, Institute of Population Health, University of Manchester, Manchester, UK; **Mel Slater**, DSc, Department of Computer Science, University College London, London, UK and Institució Catalana de Recerca i Estudis Avançats (ICREA), University of Barcelona, Spain; **David M. Clark**, DPhil, Department of Experimental Psychology, University of Oxford, Oxford, UK

## Abstract

**Background**

Persecutory delusions may be unfounded threat beliefs maintained by safety-seeking behaviours that prevent disconfirmatory evidence being successfully processed. Use of virtual reality could facilitate new learning.

**Aims**

To test the hypothesis that enabling patients to test the threat predictions of persecutory delusions in virtual reality social environments with the dropping of safety-seeking behaviours (virtual reality cognitive therapy) would lead to greater delusion reduction than exposure alone (virtual reality exposure).

**Method**

Conviction in delusions and distress in a real-world situation were assessed in 30 patients with persecutory delusions. Patients were then randomised to virtual reality cognitive therapy or virtual reality exposure, both with 30 min in graded virtual reality social environments. Delusion conviction and real-world distress were then reassessed.

**Results**

In comparison with exposure, virtual reality cognitive therapy led to large reductions in delusional conviction (reduction 22.0%, *P* = 0.024, Cohen's *d* = 1.3) and real-world distress (reduction 19.6%, *P* = 0.020, Cohen's *d* = 0.8).

**Conclusion**

Cognitive therapy using virtual reality could prove highly effective in treating delusions.

Individuals with persecutory delusions erroneously believe that others are trying to cause them physical, psychological or social harm. Our psychological conceptualisation is that at the heart of persecutory delusions are unfounded threat beliefs.^1^ One reason for the persistence of the threat beliefs is a failure to obtain and process disconfirmatory evidence as a result of the use of safety-seeking behaviours. The concept of safety-seeking behaviours was developed in cognitive accounts of anxiety.^2^ Individuals who consider themselves threatened carry out actions designed to prevent the feared catastrophe from occurring. When the judgement of a threat is unrealistic the use of safety-seeking behaviours has important consequences: individuals believe that the threat was averted by the use of the safety-seeking behaviour (for example ‘The reason I wasn't attacked was because I quickly got off the bus’, ‘I was safe because I didn't go out’) rather than conclude that the original idea was inaccurate. A number of experimental studies have evaluated safety behaviours as a maintenance factor in anxiety disorders, finding that testing out the fear cognitions by dropping safety behaviours (a key technique of cognitive therapy) leads to greater reductions in the threat beliefs than exposure methods alone.^3–7^ In this report we conduct such a test for the first time in patients with persecutory delusions.

Almost all patients with persecutory delusions report using safety-seeking behaviours.^8,9^ The most common type of safety behaviour is avoidance. For example, patients often try to minimise the number of times that they go outside the home, particularly avoiding being in enclosed public places with other people. More subtle, but equally important, within-situation behaviours occur when in the places of perceived threat. For example, patients take steps to decrease their visibility, enhance their vigilance and look out for escape routes. The target for successful treatment is for patients to relearn that they are safe and hence diminish their delusional conviction and related distress. Therefore, patients need to test out the persecutory threat beliefs by entering the feared situations and not using safety-seeking behaviours. However, many patients with persecutory delusions find it too difficult to enter their feared situations because of the intolerable anxiety generated. When they are admitted to psychiatric hospital, their opportunities for such learning are often even more restricted.

The solution we have been developing is to use virtual reality.^10^ An immersive virtual reality system creates a surrounding three-dimensional computer-generated world in which a person can physically move and interact with objects and virtual people (avatars). Virtual reality elicits responses in individuals similar to those that would occur in the real situation.^11,12^ A remarkable example is how graded exposure in virtual reality for anxiety disorders is as efficacious as exposure in the real world.^13^ Virtual social environments could provide a means for patients with severe paranoia to make the first steps towards entering their feared situations, before taking the learning into the real world. Our study was designed to test the hypothesis that persecutory delusions are threat beliefs maintained by safety behaviours, and to establish the potential therapeutic use of virtual reality for delusions. The methodology was drawn from studies of safety behaviours in anxiety disorders,^3–7^ but with virtual reality used to present the feared situations. The study was a short-term test of the use of virtual reality and was not designed as a clinical trial. It was predicted that testing the threat predictions of the delusions when not using safety behaviours (virtual reality cognitive therapy), compared with exposure alone (virtual reality exposure), would lead to gradually lower levels of paranoia and distress being experienced during the periods in virtual reality; an overall reduction in the degree of conviction with which the persecutory delusions were held; and lower distress in a real social situation.

## Method

### Participants

Thirty patients with persecutory delusions were recruited from adult mental health services in Oxford Health NHS Foundation Trust. Inclusion criteria were: a current persecutory delusion as defined by Freeman & Garety^14^ (an unfounded belief that harm is occurring, or is going to occur, to him or her and that the persecutor has the intention to cause harm); the delusion held with at least 50% conviction; a case-note diagnosis of non-affective psychosis; reporting feeling threatened when around other people and using within-situation safety behaviours. Exclusion criteria were: a primary diagnosis of alcohol or substance dependency; organic syndrome or intellectual disability (also known as learning disability in UK health services); photosensitive epilepsy; and a command of spoken English inadequate for engaging in the study. Examples of the content of the persecutory delusions included: ‘People are trying to cause me physical, mental, and emotional harm’; ‘When I go out the devil and others persecute me’; ‘People know what I'm thinking and want to kill me’; ‘People see me as an easy target and do things to belittle me’; ‘When I go out people are making derogatory comments in order to upset me’; ‘Someone intends to kill me’.

### Design

The study had a between-groups design. It had approval from a National Health Service research ethics committee and was registered on the UKCRN Portfolio database (UKCRN ID 12951). The principal testing for each patient took place in one day, beginning and ending at the patient's home (or hospital ward in two instances). Before randomisation, conviction in the persecutory delusion was rated and then the patient completed a 5 min behaviour test in which they entered a real-life social environment that they wanted to be less fearful in (for example walking to the local shop). Patients were then brought to the virtual reality laboratory and at this stage randomised to either virtual reality cognitive therapy (threat belief tests in virtual reality with the dropping of safety behaviours, the threat belief testing group) or virtual reality exposure with keeping of safety behaviours (the exposure group). Randomisation was carried out using an online generator (www.randomization.com). There were seven brief periods in virtual reality, with ratings of conviction in the delusion and related distress completed before and after each immersion.

The virtual reality exposure instructions read aloud were:

‘The best way to deal with a fear is to go into the situation. And learn that you are safer than you think. However, this is easier said than done, since going into these situations makes us anxious and that feels bad. Therefore we are going to make it easier for you. By gradually getting used to people in a computer world. First, you'll have a look at the computer world without any computer characters in it. A chance to try it out. After that we'll introduce a small number of people. And gradually build it up each time you try the virtual reality. You'll go in seven times, each lasting about five minutes. It'll give you a great chance to learn that you are safer than you feared. It works a bit like getting into cold water; when you first get in it feels uncomfortable, but after a while you get used to it, as long as you stay in. Please do use any strategies such as [add person's safety behaviours] that give you the confidence to remain in the situation.’

The virtual reality cognitive therapy instructions were the same, except the last line was replaced with:

‘But truly to learn you are safe you need to let your defences down. Find out that it isn't your defences that are keeping you safe but simply that you would be okay anyway. This can be very freeing. As you mentioned, when you are around other people you [add person's safety behaviours] and you believe that this keeps you safe. However, you need to learn what happens if you don't [add person's safety behaviours]. This is the way to find out you are truly safe. Indeed this is a great chance to try everything you can to find out you are safe. So instead of [add person's safety behaviours] you could try something quite different, almost the opposite, like [add alternative strategy]. This will make you much more confident that nothing bad is going to happen. It is a chance to discover your confidence around other people.’

After the completion of virtual reality the patient returned home, the behaviour test was repeated, and, finally, the persecutory delusion re-rated. The typical testing times were about 20 min to complete the questionnaires and behavioural test at home, about 60–90 min in the virtual reality laboratory, and then 20 min to complete the repeat behavioural test and final assessments. The periods of time in virtual reality were usually conducted one after the other but occasionally patients wanted short breaks between the environments. The length of the whole testing session depended upon the journey time by taxi between a patient's home and the virtual reality laboratory. Testing throughout the day was carried out by a clinical psychologist (J.B. or D.F.) who explained the experimental conditions and a research worker (E.B., N.D., N.E. or E.C.) who conducted the assessments.

### Assessments

A few days before the testing, participants completed the Positive and Negative Syndrome Scale – positive subscale (PANSS),^15^ the Psychotic Symptoms Rating Scale – Delusions (PSYRATS),^16^ the Safety Behaviours Questionnaire – Persecutory Beliefs,^8^ the Beck Anxiety Inventory^17^ and the Beck Depression Inventory.^18^

During the testing day the key variables (conviction and distress) were assessed using visual analogue rating scales. Each line was 100 mm long, and where the patient marked each line was recorded as a number between 0 and 100. At the beginning and end of the testing day, participants were asked to rate how strongly they believed their persecutory belief on a 0% (do not believe at all) to 100% (absolutely certain) scale. Before going into the real-world situation or into a virtual reality scenario, participants rated ‘At this moment, how convinced are you that your worries are true?’ on a 0 (not convinced at all) to 10 (absolutely certain) scale and ‘How distressed do you feel about your worries?’ on a 0 (not distressed at all) to 10 (extremely distressed) scale. These scales were explicitly linked to the persecutory concerns, and were then repeated after the real-world situation or virtual reality. An additional question was also completed, ‘How distressed did you feel going outside?’ or ‘How distressed did you feel in the virtual reality scenario?’, rated on a 0 (not distressed) to 10 (extremely distressed) scale.

A credibility rating for the two randomisation conditions was also introduced for the last 11 participants. After being given the virtual reality cognitive therapy or virtual reality exposure instructions, patients were asked to rate the question ‘How much do you believe that this will help to reduce your fears about others?’ on a 0 (not convinced at all) to 100 (absolutely certain) visual analogue scale.

### Virtual reality

Participants could walk around the laboratory room immersed in the virtual world via a head-mounted display (HMD) linked to a computer and tracking system. Full details of our laboratory equipment and the virtual reality scenarios are provided in the online supplement DS1 and Figs DS1 and DS2. There were two virtual reality environments: an underground train ride and a lift. Each had gradations of difficulty based on the number of avatars placed around where the participant could walk (see Fig. DS2). The underground scenarios progressed from no avatars being present to 22 being in the carriage. The lift scenarios progressed from two avatars being in the lift to there being six. Movement data were recorded for the underground scenarios (since there was much greater opportunity for walking compared with the confined lift space).

### Analysis

The main outcome predictions – concerning the effect of allocation to randomisation condition on delusional conviction at the beginning and end of testing and the distress in the real-world situation – were tested using analysis of covariance, controlling for initial score. These analyses were carried out using SPSS Version 20.0. All hypothesis testing was two-tailed. There were no missing data. Effect sizes were calculated using Cohen's *d*, taking the estimated coefficient of allocation from the ANCOVA divided by the pooled baseline standard deviation.

For the visual analogue ratings from the virtual reality social environments, random-effects models (to allow for correlation between measures repeated over time) looking at the effect of allocation, environment (virtual reality level) and the allocation × environment interaction, were carried out using Stata version 13.1. We tested whether there was an effect of allocation on pre-virtual reality scores, post-virtual reality scores, pre- to post-change (i.e. pre-minus post-), and the mean of the pre- and post-virtual reality scores. Although there is redundancy in this analysis strategy, we chose this sequence of analyses in order to clearly illustrate the patterns of learning indicated by the data. So, the analysis of the pre-virtual reality scores illustrates the effect of allocation condition that is carried forward from one virtual reality level to subsequent levels (we would not expect to see an allocation effect for the first virtual reality social environment (VR1) but would hope to see growing allocation effects from the second). Analysis of post-virtual reality data shows the allocation intervention effects on the combined within- and between-level differences. The analysis of the pre-minus post-virtual reality change scores looks at the effect of the allocation conditions on within-session learning. The analysis of the pre-/post-virtual reality means illustrates the effect of allocation that is common to both pre- and post-virtual reality measures.

The target sample size was 30 patients with persecutory delusions. We were expecting large effect size reductions in delusional conviction, with an approximate halving in the threat belief testing group and relative constancy in the exposure group. If, for example, the initial visual analogue scale score for conviction before going into virtual reality was 50 (s.d. = 20) then a simple *t*-test of final between-group scores, with a 0.05 two-sided significance level, would have over 90% power to detect such a large effect size (*d* = 1.25) reduction in the threat belief testing group.^19^ For comparison, the between-participants clinical anxiety disorders studies have tested 9 patients with panic disorder in each condition^4^ and 15 people with social anxiety in each condition.^6^

## Results

Basic demographic and clinical information for the participants is summarised in Table 1. Typical of studies of adult patients with current psychotic experiences, the average age is approximately 40 years old, there is a greater number of men than women, the most common clinical diagnosis was schizophrenia, hallucinations were occurring in half of the group, the overwhelming majority were unemployed, and rates of depression and anxiety were high. The randomisation condition credibility ratings were broadly comparable in the two groups, with a mean score of 43.8 (s.d. = 20.8, *n* = 6) in the threat belief testing group and a slightly higher credibility mean score of 53.8 (s.d. = 19.6, *n* = 5) in the exposure group.

**Table 1:** Demographic and clinical data by randomisation condition

Variable	Threat belief testing group (*n* = 15)	Exposure group (*n* = 15)
Age, years: mean (s.d.)	42.1 (13.4)	40.6 (14.4)

Men/women, *n*	10/5	6/9

Ethnicity, *n*		
White	14	15
Mixed	1	0

Employment status, *n*		
Unemployed	13	14
Part-time employed	1	0
Full-time employed	0	0
Volunteer	0	0
Retired	1	1
Student	0	0

Clinical diagnosis, *n*		
Schizophrenia	10	10
Schizoaffective disorder	2	1
Delusional disorder	0	2
Psychosis NOS	3	2

PSYRATS – delusions score, mean (s.d.)	17.7 (2.6)	16.9 (2.8)

PANSS – positive score, mean (s.d.)	17.5 (2.6)	17.4 (3.2)

Experiencing hallucinations (PANSS hallucination score >3), *n*	8	7

Safety Behaviour Questionnaire score, mean (s.d.)	42.3 (11.7)	36.9 (14.6)

Depression (BDI), *n*		
None (0–13)	1	4
Mild (14–19)	1	1
Moderate (20–28)	4	3
Severe (29–63)	9	7

Anxiety (BAI), *n*		
None (0–7)	0	1
Mild (8–15)	1	1
Moderate (16–25)	5	4
Severe (26–63)	9	9

NOS, not otherwise specified; PSYRATS, Psychotic Symptoms Rating Scale; PANSS, Positive and Negative Syndrome Scale; BDI, Beck Depression Inventory; BAI, Beck Anxiety Inventory.

### Ratings in virtual reality

Table 2 provides a summary of the movement data and ratings of paranoia conviction and distress during the virtual reality testing. The full results of the random-effects models are provided in online supplement DS2. For total movement in virtual reality, it can be seen that the two groups moved similarly in the empty carriage, but that the threat belief testing group began to move more in the social environments, consistent with the instructions of dropping safety behaviours and exploring the environment fully. This was confirmed in a random-effects model, with no significant difference in movement between the two groups in the empty carriage (coefficient 0.9, s.e. = 3.7, *P* = 0.804), but an additional 10.5 metres movement in the final virtual reality underground level by the threat belief testing group compared with the exposure group (s.e. = 3.0, *P*<0.001).

**Table 2:** Visual analogue scale ratings for the virtual reality testing

	Mean (s.d.)	
Variable	Threat belief testing group (*n* = 15)	Exposure group (*n* = 15)
Total movement (m) in:		
Empty train	17.4 (6.9)	17.1 (7.4)
VR1	23.5 (7.7)	19.0 (8.3)
VR2	36.1 (12.7)	24.1 (9.7)
VR3	37.0 (13.4)	25.5 (12.2)

Conviction before:		
VR1	65.6 (27.9)	65.7 (24.8)
VR2	57.2 (29.6)	70.7 (21.4)
VR3	56.2 (27.0)	74.7 (19.9)
VR4	55.5 (26.8)	72.0 (22.2)
VR5	50.7 (29.2)	71.3 (22.1)
VR6	47.3 (28.3)	69.5 (22.9)

Conviction after:		
VR1	55.8 (28.5)	67.9 (21.9)
VR2	56.5 (27.8)	71.7 (24.4)
VR3	50.7 (29.8)	75.2 (17.4)
VR4	47.7 (29.1)	67.0 (24.4)
VR5	46.9 (28.0)	67.5 (24.3)
VR6	45.2 (27.4)	70.9 (24.6)

Delusion distress before:		
VR1	61.6 (30.1)	59.6 (19.7)
VR2	53.5 (48.3)	61.3 (22.7)
VR3	48.3 (29.0)	67.2 (20.2)
VR4	49.8 (26.0)	66.3 (19.4)
VR5	45.7 (25.6)	59.1 (22.4)
VR6	40.9 (25.5)	57.7 (25.1)

Delusion distress after:		
VR1	53.8 (25.8)	60.0 (18.5)
VR2	52.0 (26.3)	71.7 (18.9)
VR3	45.7 (28.8)	68.1 (17.6)
VR4	45.5 (27.4)	56.1 (19.6)
VR5	45.1 (25.3)	54.6 (24.1)
VR6	40.1 (27.4)	60.4 (22.8)

Distress of:		
Empty train	33.1 (29.1)	35.5 (24.4)
VR1	41.8 (29.2)	40.3 (26.2)
VR2	49.1 (25.6)	61.1 (33.8)
VR3	37.9 (29.5)	59.3 (30.9)
VR4	36.1 (29.3)	33.1 (26.7)
VR5	39.4 (25.0)	40.1 (31.2)
VR6	38.9 (27.9)	42.0 (32.9)

VR1–6, virtual reality scenario level 1–6.

For ratings of conviction in paranoia, a gradual reduction across the scenarios for the threat belief testing group can be seen, whereas the conviction scores remain stable in the exposure group. There was no group difference in conviction prior to the first virtual reality social environment (coefficient −0.1, s.e. = 9.4, *P* = 0.994), but significant reductions in pre-virtual reality conviction ratings for the threat belief testing group compared with the exposure group with the successive times in virtual reality, so that by the final virtual reality scenario there has been an added 20.9% point reduction in conviction for the threat belief testing group compared with the exposure group (s.e. = 5.7, *P*<0.001). There was a large but non-significant reduction in conviction after the first virtual reality social environment for the threat belief testing group compared with the exposure group (coefficient −12.1, s.e. = 9.5, *P* = 0.205), which gradually increased with time, so that there was an additional 12.9% point reduction in delusional conviction for the threat belief testing group compared with the exposure group by the end of the final virtual reality scenario (s.e. = 6.3, *P* = 0.039). There was an effect of the threat belief testing allocation on the pre- to post-virtual reality reduction (within-session change) in conviction for the first virtual reality social environment (coefficient 9.7, s.e. = 4.5, *P* = 0.031) but no interactions of allocation with environment (level). The pre–post change in conviction (common to all six virtual reality scenarios) is on average 3.9% points greater in the threat belief testing group compared with the exposure group. The absence of an allocation × level interaction for the pre–post change scores indicates that the pre- and post-measurements are changing in parallel. A mixed model using the mean of the pre- and post-virtual reality scores shows a small but not significant reduction in conviction for the threat belief testing group compared with exposure after the first virtual reality social environment (coefficient −5.1, s.e. = 9.3, *P* = 0.582) but significant allocation × level interactions, so that the added reduction in conviction for the threat belief testing group relative to the exposure group by the final level is 17.9% points (s.e. = 5.0, *P*<0.001).

A very similar pattern can be seen for the distress associated with the paranoia: a gradual reduction in the threat belief testing group and relative stability in the exposure group. The results for the random-effects models mirror those seen above (see online supplement DS2). Taking the mean of the pre- and post-paranoia distress scores, there is an initial very small non-significant decrease in paranoia distress in the threat belief testing group compared with the exposure group (coefficient −0.8, s.e. = 8.7, *P* = 0.926), but significant time × level interactions, so that by the last virtual reality scenario there is an added reduction in distress of 17.6 points in the threat belief testing group compared with the exposure group (s.e. = 5.3, *P* = 0.001).

### Ratings of the delusion

The conviction level in the delusion for the threat belief testing group reduced from 79.8% (s.d. = 16.4, *n* = 15) at the beginning of the testing session to 46.5% (s.d. = 29.2, *n* = 15) at the end. For the exposure group, the conviction level in the delusion was 78.5% (s.d. = 17.1, *n* = 15) at the beginning of the testing session and 67.6% (s.d. = 25.5, *n* = 15) at the end. Assessed by ANCOVA, compared with exposure, virtual reality cognitive therapy (threat testing with the dropping of safety behaviours) led to a reduction in conviction in the delusion of 22.0% (s.e. = 9.2), 95% CI 3.2–40.9%, *F*(2,27) = 5.75, *P* = 0.024, *d* = 1.3.

### Ratings in the behaviour test

Ratings for the real-world behaviour test are displayed in Table 3. It can be seen that the scores for the first real-world test are comparable across the two groups. At the repeat, it can be seen that the threat belief testing group found the real-world task less distressing than the exposure group. Compared with virtual reality exposure, and controlling for the level of distress caused by the real-world situation the first time of entering, virtual reality cognitive therapy led to a reduction in distress in the real-world situation of 19.6% (s.e. = 7.9), 95% CI 3.4–35.7, *F*(2,27) = 6.15, *P* = 0.020, *d* = 0.8.

**Table 3:** Ratings for the real-life situation

	Mean (s.d.)	
Variable	Threat belief testing group (*n* = 15)	Exposure group (*n* = 15)
Before going outside the first time		
Conviction	78.2 (20.7)	77.1 (17.2)
Delusion distress	71.9 (22.7)	62.8 (14.9)

After going outside the first time		
Conviction	78.3 (21.8)	80.2 (15.6)
Delusion distress	76.9 (24.0)	68.8 (19.6)
Distress when outside	66.9 (29.4)	62.1 (17.1)

Before going outside the second time		
Conviction	48.7 (28.0)	69.3 (23.1)
Delusion distress	43.9 (24.6)	59.3 (21.6)

After going outside the second time		
Conviction	42.3 (27.2)	64.2 (20.8)
Delusion distress	39.6 (23.3)	53.1 (25.2)
Distress when outside	31.6 (24.2)	49.3 (22.0)

## Discussion

### Main findings

In this study it has been shown that virtual reality can be used to present computerised versions of commonly feared situations to patients with persecutory delusions; that new learning can then take place; and, importantly, that the learning transfers into the real world. The best learning was shown to occur when the persecutory beliefs were more fully put to the test by discouraging the use of safety behaviours, which are a central maintenance factor proposed in cognitive models of clinical disorders. With this type of cognitive treatment approach, patients learned that they were safer than they had feared. This is good evidence that persecutory delusions are unfounded threat beliefs maintained by safety-seeking behaviours. Paranoid thinking with brief virtual reality cognitive therapy not only reduced across the periods in virtual reality but transferred to a rating of the overall delusion and the experience of an important real-life social situation. The improvement found was over and above that of using exposure, which itself is a credible treatment technique for unfounded fears. Thus, the *in toto* therapeutic effect of virtual reality cognitive therapy, especially if the treatment is lengthened, may be even greater when compared with a placebo intervention.

### Implications

A key task in the treatment of clinical paranoia is to help patients learn that the environment is now safe for them,^20^ but this has rarely been the topic of experimental research. The underlying rationale of the current study is that avoidance of other people needs to be reduced so that patients experience directly that they are not attacked and, perhaps most importantly, that anxious feelings are tolerable. This is identical to the successful treatment of anxiety disorders.^21^ It can be very difficult for patients to approach their feared situations, especially when deliberately letting down long-built defences. Even in virtual reality the anxiety generated and effort required of the patients was plain to see. The use of virtual reality social environments may become an important treatment step in recovery from paranoia, and could prove as efficacious as has been shown for anxiety disorders.^13^ Virtual reality facilities could have a central place in mental health clinics and wards of the future.

Safety-seeking behaviours were identified using the Safety Behaviours Questionnaire – Persecutory Beliefs.^8^ It asks for ‘any actions or behaviours that you may do to try to minimize or stop the threat from occurring; often we find that individuals who feel threatened do things that they think will provide some protection’. Cognitive approaches need to be carefully tailored to the precise threat prediction and the associated safety-seeking behaviours in order to set-up an appropriate learning experience. The most common within-situation safety-seeking behaviours used by study patients were avoiding eye-gaze and keeping a distance from other people. In virtual reality cognitive therapy, patients were encouraged to do the opposite. Patients were asked to think of an alternative behaviour (for example looking at the avatars directly, getting close to them) that would really help them to gain confidence that they were safer than feared. Virtual reality social environments have the advantage that it is possible to act rather differently than is possible in real situations – indeed, an element of humour for patients can sometimes be introduced by exaggerating the alternative strategies (for example going toe-to-toe to the avatars, holding a long stare). A further advantage is that much harder social situations than typically faced by patients in daily life can be used, which can really help boost confidence.

### Limitations

There are limitations to the study. We did not determine whether there were benefits associated with the exposure condition. A third arm to the randomisation, testing time spent in non-social virtual reality environments, would have been needed. Neither the patients nor the researchers were masked to the randomisation allocations, which may have introduced bias. However, the credibility ratings from the patients for the two conditions were comparable, indicating similar expectancy for good outcomes. Further, the scores for the two groups before their first period in a virtual reality social environment were comparable but then gradually declined in the virtual reality cognitive therapy group, indicating that progressive learning occurred. A further limitation was that we could only test the effects of encouraging the dropping of safety behaviours. There are no means to ensure that the instructions were followed, with only the movement data providing unbiased corroborative evidence. The length of time spent in virtual reality was brief and the period covered by the delusion assessment limited; a clinical trial could evaluate the effects over weeks and months of a longer time spent learning in virtual reality. The introduction of a greater number of scenarios, with tailoring to the individual, would likely add to the clinical benefits. Testing in portable and less costly virtual reality hardware will clearly be of value. Relatively high-quality virtual reality is now available as a consumer product. There is considerable work to do both in testing optimal ways to reduce persecutory delusions and in harnessing technological innovations.
